# Effects of *in ovo* feeding of vitamin C on embryonic development, hatching process, and chick rectal temperature of broiler embryos

**DOI:** 10.3389/fvets.2024.1505801

**Published:** 2025-01-07

**Authors:** Shan Du, Jianchuan Zhou, Xiang Ao, Yufei Zhu

**Affiliations:** ^1^Techlex Food Co., Ltd., Chengdu, China; ^2^School of Life Science and Engineering, Southwest University of Science and Technology, Mianyang, China; ^3^DAYU Bioengineering (Xi'an) Industrial Development Research Institute, Xi’an, China; ^4^Shanxi Dayu Bioengineering Co., Ltd., Yuncheng, China

**Keywords:** vitamin C, *in ovo* feeding, embryonic development, hatching time, rectal temperature

## Abstract

Maternal nutritional status plays a crucial role in embryonic development and has persistent effects on postnatal chicks. Vitamin C (VC) plays an important role in embryonic and postnatal development involved in nutri-epigenetics. The present study was conducted to investigate the effects of *in ovo* feeding (IOF) of VC on embryonic development, egg hatching time, and chick rectal temperature. Trial 1 was conducted under normal incubation conditions without the IOF procedure and was designed to analyze the characteristics of embryonic development and establish the scoring standards for yolk absorption and the rupture of the shell membrane. The results showed that the relative weight of the embryo and residual yolk and the organ indexes were reliable indicators of embryonic development. Yolk absorption was scored 0, 1, 2, 3, and 4, with a higher score indicating more complete absorption. In addition, the rupture of the shell membrane was divided into two cases: YES and NO. Trial 2 included three groups, control (CON), normal saline (NS), and vitamin C (VC), and was designed to detect the effects of IOF of VC on the indicators in trial 1, as well as the plasma biochemical indicators. At embryonic age 11 (E11), each egg in the CON group was non-injected, each egg in the NS group was injected with 0.1 mL of sterile normal saline, and each egg in the VC group was injected with 0.1 mL of sterile normal saline containing 3 mg vitamin C. The whole day of E21 was evenly divided into three time periods: early (incubation hours 480–488), middle (incubation hours 488–496), and late (incubation hours 496–504). Among the CON, NS, and VC groups, the percentages of the early-hatched chicks (egg hatching time) were 29.31, 12.00, and 33.90%, respectively. The proportions of early and middle hatched chicks in these groups were 51.72, 42.00, and 38.27%, respectively. The rectal temperature of chicks was lower (*p* < 0.05) in the VC group than in the CON and NS groups. Compared to the NS group, the plasma biochemical indicators in the VC group showed significantly lower levels of alkaline phosphatase (ALP), total protein (TP), albumin (ALB), GLB, total bilirubin (TBIL), TBA, uric acid (UA), high-density lipoprotein cholesterol (HDL-C), and corticosterone (CORT) (*p* < 0.05). Additionally, alanine aminotransferase (ALT) had an increasing trend (*p* = 0.059) in the VC group. In conclusion, our data demonstrated that VC accelerated the hatching process and reduced chicks’ rectal temperature, which may be related to the improvement of liver function and changes in metabolism, as indicated by blood biochemical indicators.

## 1 Introduction

The developmental origins of health and disease (DOHaD) theory suggests that factors present during the gestational and early-postnatal period (nutrition, climate, stress, toxins, and exercise) can significantly influence adult health and likelihood of developing chronic diseases later in life (1). Growing evidence has shown that an impoverished *in utero* environment may cause many diseases after birth, such as cardiovascular disease, metabolic syndrome, obesity, and diabetes (2). As with livestock animals, the abovementioned effects directly reduce production performance and lead to economic losses. Malnutrition during incubation (achieved by removing quantities of albumen from the egg) can lead to low hatch rates in chicks and decreased post-hatch body weight up to 7 days of age (3). Using Arbor Acres broilers as an example, the incubation period accounts for up to one-third of the entire life cycle, and this proportion is expected to increase with the development of breeding, which will lead to the embryonic environment playing a more prominent role in the production performance of broilers.

Nutrition is an important factor affecting embryonic development and also serves as an intervention strategy that neutralizes the negative effects of other factors on embryonic development (4). Prenatal epigenetic diets, including vitamin C (VC) from fruits, resveratrol from grapes, and isothiocyanates from broccoli, help protect against environmental pollution and improve fetal and offspring health outcomes (5). Embryos derive nutrients from the mother through the placenta in mammals, while avian embryos only rely on the nutrients deposited in the eggs. If there is insufficient nutrient deposition in breeder eggs, it may directly affect embryonic development and the offspring’s production performance. Our previous studies reported that the endogenous synthetic ability of VC was weak during embryonic development, and VC supply was mainly from endogenous absorption from the yolk and albumen (6). However, dietary VC supplementation failed to increase VC deposition in the yolk and albumen (7). In addition, stress during the breeding process reduced the synthesis capacity of VC, and VC was preferentially used to eliminate stress at the same time (8), leading to less deposition in breeder eggs.

VC plays an important role in embryonic or postnatal development, and vitamin C supplementation during pregnancy improves placental function (9). Some studies have shown that *in ovo* feeding (IOF) of VC has positive effects on growth performance in postnatal broilers (10), suggesting that VC might improve embryonic development in broilers based on the DOHaD theory. In addition, the elimination and reconstruction of DNA methylation occur during embryonic development in broilers (11), and VC can act as a cofactor for related enzymes involved in DNA and histone demethylation (12).

The hatching process is closely related to embryonic development and is also reflected in the rupture of the shell membrane (or shell), yolk absorption, and hatching time (13). In addition, rectal temperature is a reliable indicator for evaluating a chick’s stress resistance both inside and outside of the egg (14). In this study, we investigated the effects of IOF of VC on embryonic development, the hatching process, and the rectal temperature of newly hatched chicks. We also explored the potential underlying mechanisms.

## 2 Materials and methods

The animal experimental procedures were approved by the Institutional Animal Care and Use Committee of Southwest University of Science and Technology (Permit Number: L2023013).

### 2.1 Broiler breeder flocks, egg collection, and incubation

At embryonic age 11 (E11), the eggs were candled, and the unfertilized eggs and dead embryos were removed. At E19, all the eggs were transferred to hatching baskets. All the eggs, sourced from Arbor Acres broiler breeder flocks, were purchased from the Xianyang Dacheng Poultry Industry Co. Ltd. The fertilized eggs were collected from the same flock at 27 weeks of age for trial 1 and 31 weeks of age for trial 2. All the eggs were incubated in an automatic incubator, and the hatching program was set to chicken mode.

#### 2.1.1 Trial 1

A total of 300 disinfected eggs were randomly divided into 10 replicates, with 30 eggs per replicate. Each egg was weighed, and its weight was recorded on the eggshell. At E19, each egg was put into a mesh bag. At E11, E13, E15, E17, E19, E20, E21, and on the postnatal 1st day (D1), one egg or chick per replicate was selected for the measurement of embryonic or chick weight and organ indexes. Every 2 h, starting from E19.5 to E20.5, one egg per replicate was selected for the standard establishment of yolk absorption and shell membrane rupture scores. The eggs were incubated using an automatic incubator (9TV-2A, Beijing Blue Sky Electronic Technology Co., Ltd.). The incubation procedures were as follows: (1) E1–E6: temperature 38°C, humidity 60%; (2) E7–E12: temperature 37.8°C, humidity 55%; (3) E13–E18: temperature 37.6°C, humidity 60%; and (4) E19–E21: temperature 37.2°C, humidity 70%. The eggs were turned every 2 h before E19 and not turned after E19.

#### 2.1.2 Trial 2

Two incubators (A and B) were used in trial 2. Three groups were set up in both incubators A and B: control (CON), normal saline (NS), and vitamin C (VC) groups. At E11, each egg in the CON group was non-injected, each egg in the NS group was injected with 0.1 mL of sterile normal saline, and each egg in the VC group was with injected 0.1 mL of sterile normal saline containing 3 mg of vitamin C. The injection site was the yolk sac. The egg injection method is based on a previous publication (15). A total of 450 disinfected eggs were randomly divided into three groups with 10 replicates in incubator A, while a total of 210 disinfected eggs were randomly divided into three groups without replicates in incubator B. At E13, E15, E17, E19, E20, E21, and on D1, one egg or chick per replicate was selected for the measurement of embryonic or chick weight and organ indexes in incubator A. At the end of E20, two eggs per replicate were selected for yolk absorption and shell membrane rupture scoring in incubator A. After hatching, two chicks per replicate were selected for the measurement of rectal temperature and then one chick per replicate was selected for plasma collection in incubator A. All the eggs were used for analyzing the hatching time in incubator B.

### 2.2 Embryonic weight and organ indexes

After the embryo or chick was slaughtered, the weight of the embryo or chick, residual yolk, liver, heart, lung, and gallbladder was measured. The weight of the embryo or chick and residual yolk was expressed as the ratio of each to the egg weight. The organ index was expressed as the ratio of the organ weight to the embryonic weight, excluding the residual yolk.

### 2.3 Yolk absorption and the rupture of the shell membrane score

To the best of our knowledge, this was the first time a standard for yolk absorption and shell membrane rupture scoring was established. The egg was opened from the air chamber, and the status of the rupture of the shell membrane was photographed. Then, the embryo was immediately slaughtered and placed in a Petri dish, and the status of yolk absorption was photographed. In the end, a visual scoring standard was established. At a certain time, all the eggs to be evaluated were removed from the incubator, and the evaluation was completed within 30 min.

### 2.4 Rectal temperature and plasma parameters in the newly hatched chicks

After the feathers of the newly hatched chicks were dry, the rectal temperature was measured. The plasma biochemical parameters included alanine aminotransferase (ALT), aspartate aminotransferase (AST), alkaline phosphatase (ALP), total protein (TP), albumin (ALB), globulin (GLO), total bilirubin (TBIL), blood urea nitrogen (BUN), uric acid (UA), creatinine (CRE), glucose (GLU), total cholesterol (TC), total triglyceride (TG), high-density lipoprotein cholesterol (HDL-C), and low-density lipoprotein cholesterol (LDL-C). They were detected using an automated biochemical analyzer (Hitachi, Tokyo, Japan). The plasma samples were sent to the laboratory department of Mianyang Central Hospital (Mianyang, China) to measure the levels of thyroxin (T4), free thyroid hormone (fT4), triiodothyronine (T3), and free triiodothyronine hormone (fT3) using electro-chemiluminescence immunoassay. The plasma corticosterone (CORT) level was measured using the Chicken Corticosterone ELISA Kit (Shanghai Enzyme Biotechnology Co. Ltd., Shanghai, China).

### 2.5 Hatching time

**Figure 1:**
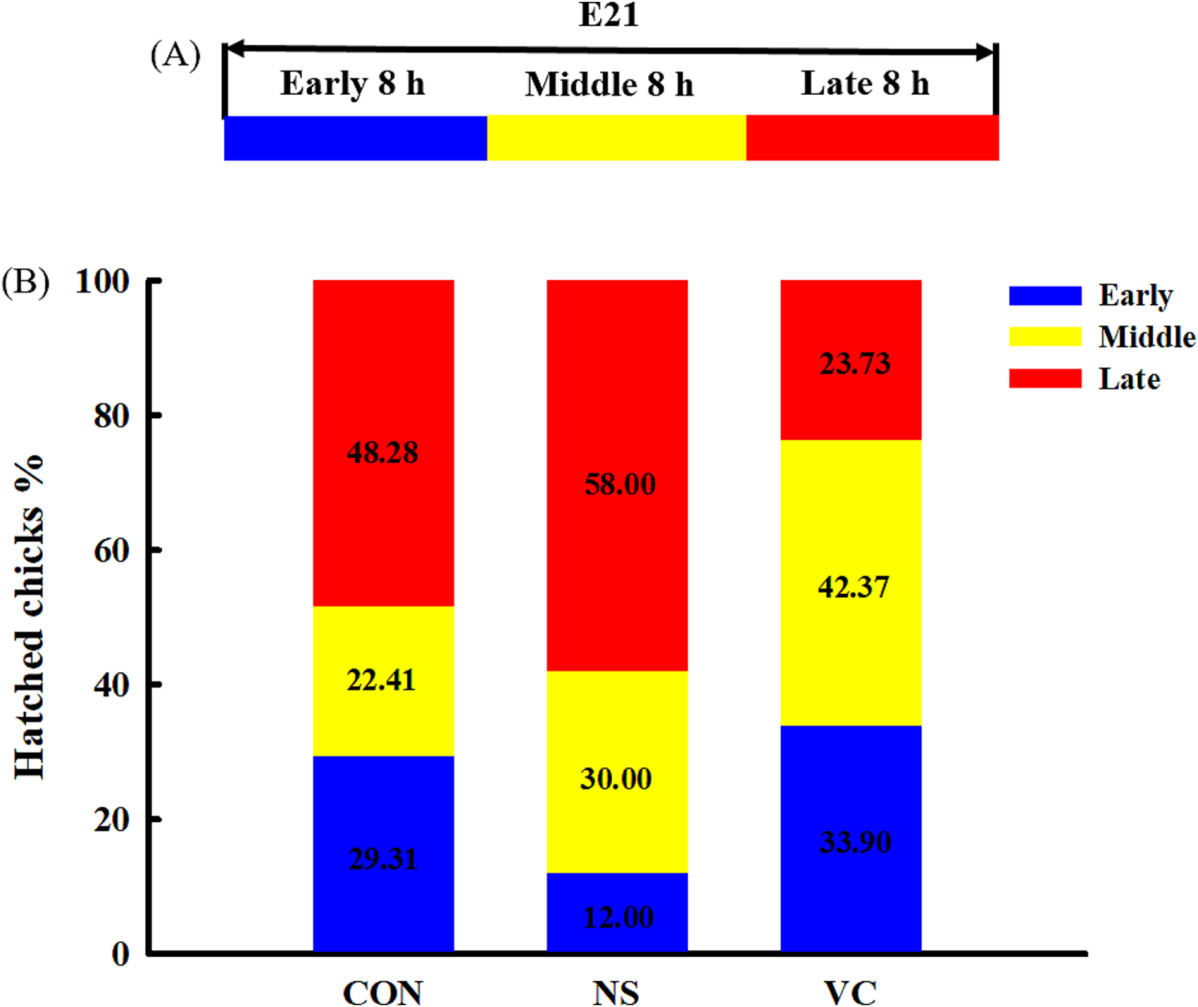
Three stages of analyzing hatchability **(A)** and effects of *in ovo* feeding of vitamin C on egg hatching time **(B)**.

As shown in Figure 1A, the entire day of E21 was evenly divided into three time periods: early (incubation hours 480–488), middle (incubation hours 488–496), and late (incubation hours 496–504). The hatched chicks in each group were counted individually during the early, middle, and late periods. These periods were calculated as a percentage of the total number of the hatched chicks for each group.

### 2.6 Statistical analysis

The data on the embryonic or chick weight, organ indexes, and chick rectal temperature were analyzed using one-way ANOVA, and the data on the plasma parameters were analyzed using an independent sample *t-*test with SPSS 21.0 (SPSS Inc., Chicago, IL, United States). Statistical significance was considered at *p* < 0.05 and trends at *p* < 0.1. All other data were compared in absolute value.

## 3 Results

### 3.1 Embryonic development characteristics

**Figure 2:**
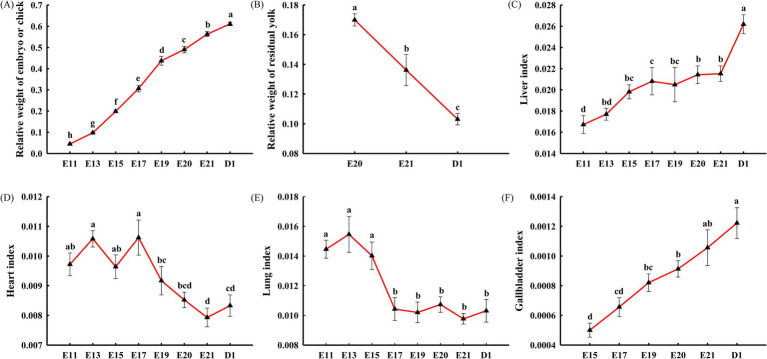
Embryonic development characteristics during incubation in the broiler chickens, including the relative weight of the embryo or chick **(A)**, the relative weight of the residual yolk **(B)**, the liver index **(C)**, the heart index **(D)**, the lung index **(E)**, and the gallbladder index **(F)**. Each triangle represents the mean ± SEM (*n* = 10). Triangles with different letters are significantly different (*p* < 0.05). The relative weight of the embryo or chick refers to the ratio of the weight of the embryo or chick, excluding the residual yolk, to the initial egg weight. The relative weight of the residual yolk refers to the ratio of the weight of the residual yolk to the initial egg weight. The organ index refers to the ratio of the organ weight to the embryonic or chick’s weight, excluding the residual yolk.

As shown in Figure 2A, the relative weight of the embryo or chick gradually increased from E11 to D1 (*p* < 0.05). The relative weight of the residual yolk gradually decreased from E20 to D1 (*p* < 0.05, Figure 2B). As shown in Figures 2C,F, the liver and gallbladder indexes both reached the maximum on D1 (*o* < 0.05). The liver index showed no significant difference at E15, E17, E19, E20, and E21 (*p* > 0.05, Figure 2C), and the gallbladder index showed no significant difference at E19, E20, and E21 (*p* > 0.05, Figure 2F). As shown in Figure 2D, the heart index showed no significant difference at E11, E13, E15, and E17 and at E20, E21, and D1 (*p* > 0.05), but the heart index was significantly higher at E11, E13, E15, and E17 than at E21 and on D1 (*p* < 0.05). As shown in Figure 2E, the lung index showed no significant difference at E11, E13, and E15 and at E17, E19, E20, E21, and D1 (*p* > 0.05), and the lung index at E11, E13, and E15 was significantly higher than that at E17, E19, E20, E21, and D1 (*p* < 0.05).

### 3.2 Standard of yolk absorption and the rupture of the shell membrane score

**Figure 3:**
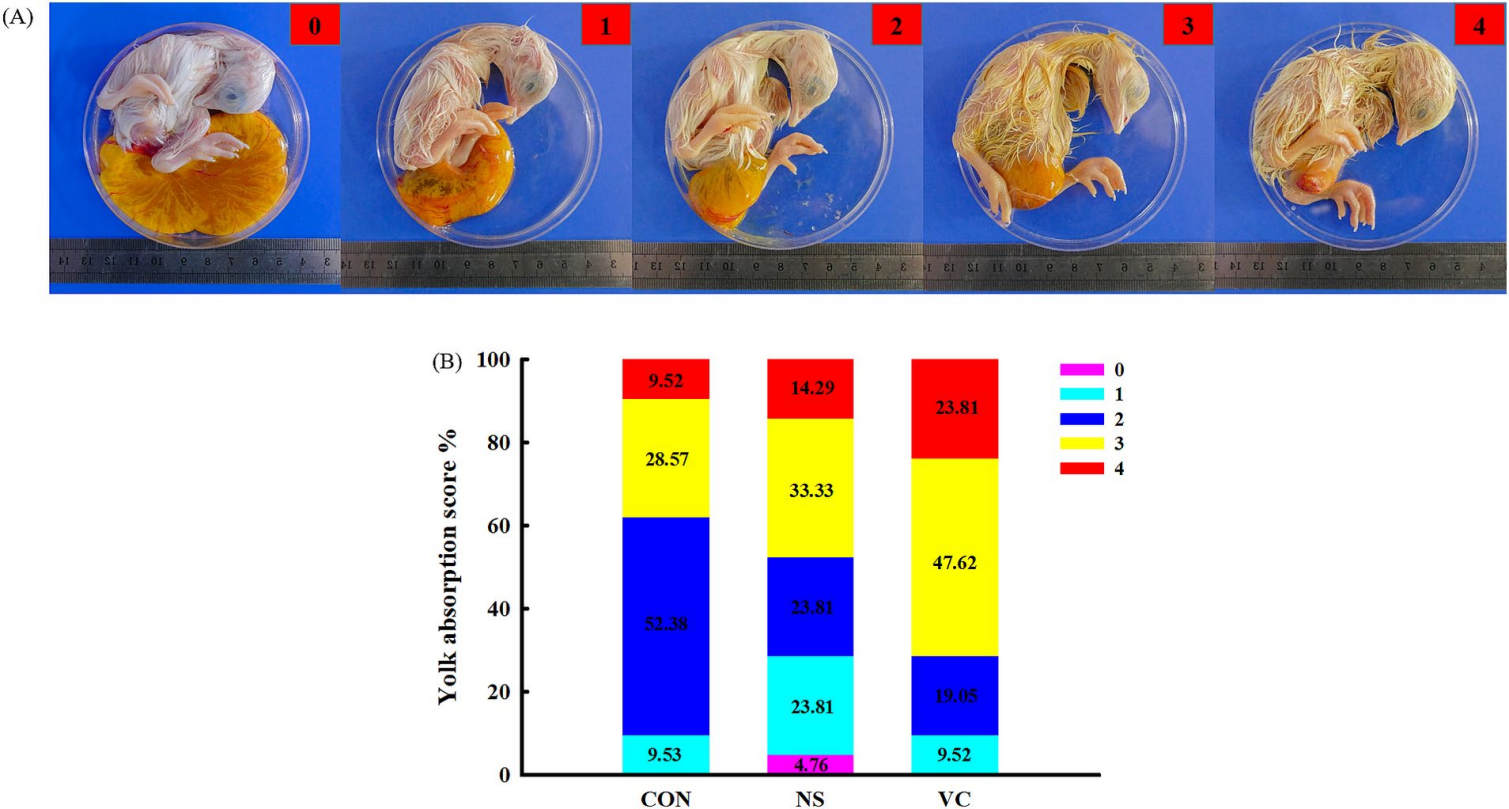
Standard of the yolk absorption score **(A)** and effects of *in ovo* feeding of vitamin C on the yolk absorption score **(B)**.

**Figure 4:**
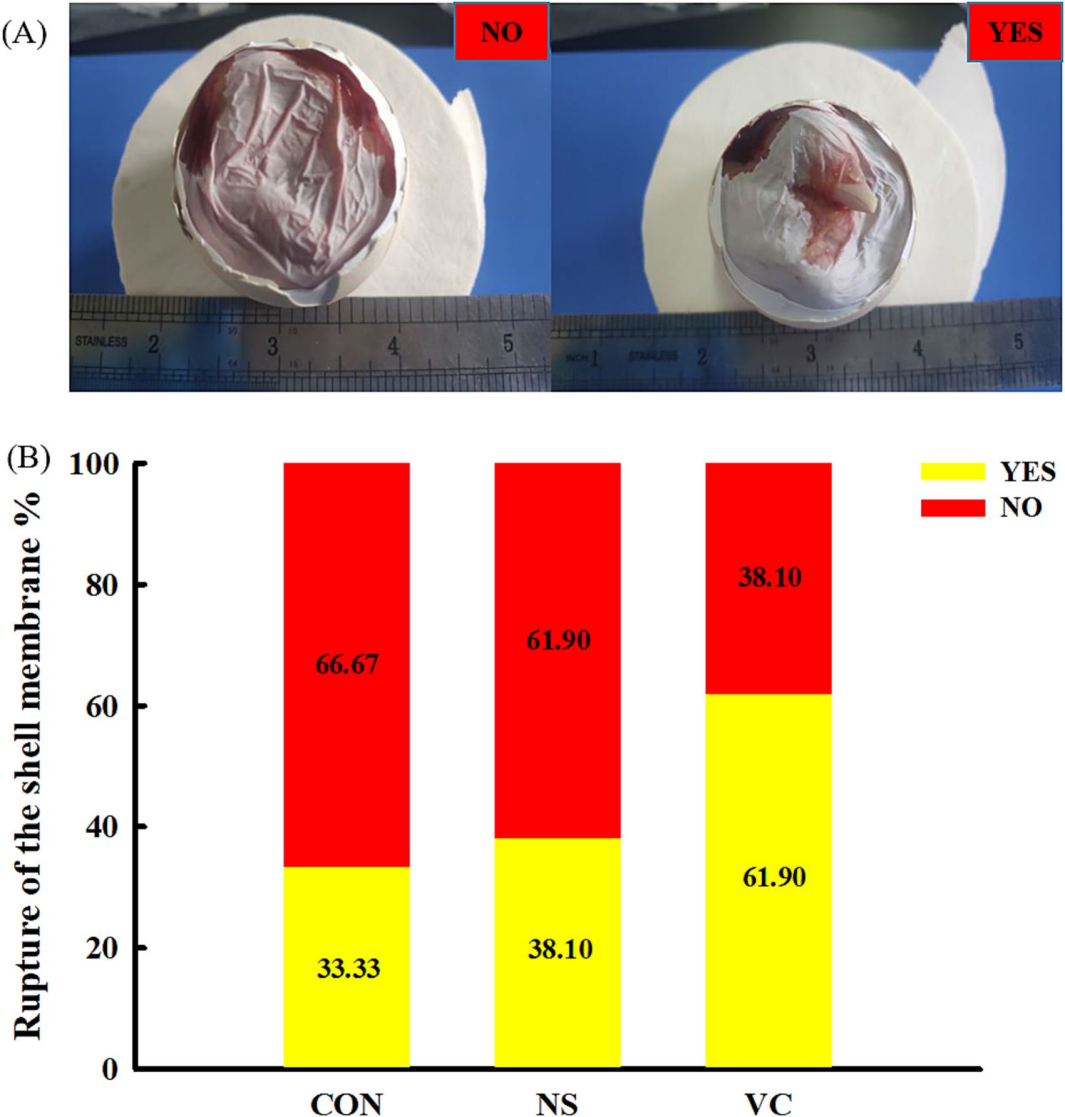
Standard of the rupture of the shell membrane score **(A)** and effects of *in ovo* feeding of vitamin C on the yolk absorption score **(B)**.

As shown in Figure 3A, yolk absorption was divided into five representative cases, which were scored 0, 1, 2, 3, and 4. The higher the score, the more complete the yolk absorption. As shown in Figure 4A, the rupture of the shell membrane was divided into two cases: YES and NO. YES meant that the shell membrane had been ruptured, while NO meant the opposite.

### 3.3 Effect of vitamin C on the embryonic development characteristics

As shown in Table 1, IOF of VC had no significant influence on the relative weight of the embryo or chick at E13, E15, E17, E19, E20, E21, and on D1 and the relative weight of the residual yolk at E20, E21 and on D1 (*p* > 0.05). As shown in Table 2, IOF of VC had no significant influence on the organ indexes of the heart, liver, lung, and gallbladder (except at E13) at E13, E15, E17, E19, E20, E21, and on D1 (*p* > 0.05).

**Table 1:** Effects of *in ovo* feeding of vitamin C at E11 on the relative weight of the embryo or chick and the residual yolk during incubation in broiler chickens.

Times	Items	Treatments			SEM	*p*-value
		CON	NS	VC		
E13	Weight of the embryo	5.76	5.60	5.86	0.095	0.553
	Relative weight of the embryo	0.098	0.094	0.099	0.0018	0.570
E15	Weight of the embryo	11.98	11.68	12.15	0.217	0.686
	Relative weight of the embryo	0.198	0.200	0.203	0.0038	0.899
E17	Weight of the embryo	18.25	19.88	18.17	0.398	0.135
	Relative weight of the embryo	0.306	0.327	0.302	0.0065	0.251
E19	Weight of the embryo	25.78	26.46	26.87	0.483	0.664
	Relative weight of the embryo	0.437	0.438	0.444	0.0078	0.931
E20	Weight of the embryo	29.16	30.46	30.18	0.352	0.294
	Relative weight of the embryo	0.490	0.456	0.507	0.0176	0.510
	Weight of the residual yolk	10.13	9.88	9.99	0.210	0.895
	Relative weight of the residual yolk	0.170	0.165	0.167	0.0030	0.808
E21	Weight of the embryo	33.644	35.312	34.065	0.355	0.136
	Relative weight of the embryo	0.563	0.582	0.570	0.0049	0.257
	Weight of the residual yolk	7.99	8.02	7.62	0.254	0.780
	Relative weight of the residual yolk	0.136	0.132	0.127	0.0040	0.680
D1	Weight of the embryo	36.65	38.04	36.39	0.427	0.253
	Relative weight of the chick	0.611	0.626	0.612	0.0052	0.463
	Weight of the residual yolk	6.20	5.54	6.27	0.201	0.282
	Relative weight of the residual yolk	0.103	0.091	0.106	0.2006	0.179

E13, embryonic age 13; D1, postnatal day 1; CON, the non-injected group; NS, the normal saline group; and VC, the vitamin C group. Relative weight of the embryo or chick, the ratio of the weight of the embryo or chick without the residual yolk to the initial egg weight. Relative weight of the residual yolk, the ratio of the weight of the residual yolk to the initial egg weight.

**Table 2:** Effects of *in ovo* feeding of vitamin C at E11 on the embryonic or chick organ indexes of the heart, liver, lungs, and gallbladder during incubation in broiler chickens.

Times	Items	Treatments			SEM	*P*-value
		CON	NS	VC		
E13	Heart	0.011	0.011	0.011	0.0002	0.759
	Liver	0.018	0.019	0.018	0.0003	0.162
	Lungs	0.015	0.015	0.015	0.0005	0.882
E15	Heart	0.010	0.010	0.010	0.0002	0.733
	Liver	0.020	0.018	0.020	0.0004	0.830
	Lungs	0.014	0.014	0.014	0.0004	0.942
	Gallbladder	0.0005	0.0005	0.0005	0.00002	0.104
E17	Heart	0.011	0.010	0.011	0.0002	0.786
	Liver	0.021	0.022	0.022	0.0005	0.534
	Lungs	0.010	0.011	0.012	0.0003	0.222
	Gallbladder	0.0007	0.0007	0.0007	0.00003	0.992
E19	Heart	0.009	0.009	0.009	0.0002	0.289
	Liver	0.020	0.021	0.022	0.0007	0.728
	Lungs	0.010	0.012	0.011	0.0004	0.462
	Gallbladder	0.0008	0.0009	0.0008	0.00003	0.577
E20	Heart	0.009	0.008	0.009	0.0002	0.820
	Liver	0.021	0.022	0.021	0.0004	0.970
	Lungs	0.011	0.010	0.010	0.0003	0.600
	Gallbladder	0.0009	0.0010	0.0011	0.00005	0.419
E21	Heart	0.008	0.007	0.008	0.0002	0.452
	Liver	0.022	0.020	0.020	0.0004	0.255
	Lungs	0.010	0.009	0.009	0.0003	0.576
	Gallbladder	0.0011	0.0010	0.0009	0.00005	0.528
D1	Heart	0.008	0.008	0.008	0.0002	0.387
	Liver	0.026	0.024	0.024	0.0005	0.062
	Lungs	0.010	0.010	0.010	0.0004	0.816
	Gallbladder	0.0012	0.0012	0.0014	0.00007	0.465

E13, embryonic age 13; D1, postnatal day 1; CON, the non-injected group; NS, the normal saline group; and VC, the vitamin C group. Organ index, the ratio of the organ weight to the embryonic or chick’s weight without the residual yolk.

### 3.4 Effect of vitamin C on the yolk absorption score and the rupture of the SHELL membrane

As shown in Figure 3B, the proportions of a score of 4 among the CON, NS, and VC groups were 9.52, 14.29, and 23.81%, respectively; the proportions of scores 3 and 4 among the CON, NS, and VC groups were 38.09, 47.62, and 71.43%, respectively; and the proportions of scores 0, 1, and 2 among the CON, NS, and VC groups were 61.91, 52.38, and 28.57%, respectively. As shown in Figure 4B, the proportions of YES among the CON, NS, and VC groups were 33.33, 38.10, and 61.90%, respectively, while the proportions of NO among the CON, NS, and VC groups were 66.67, 61.90, and 38.10%, respectively.

### 3.5 Effect of vitamin C on the egg hatching time and chick rectal temperature

**Figure 5:**
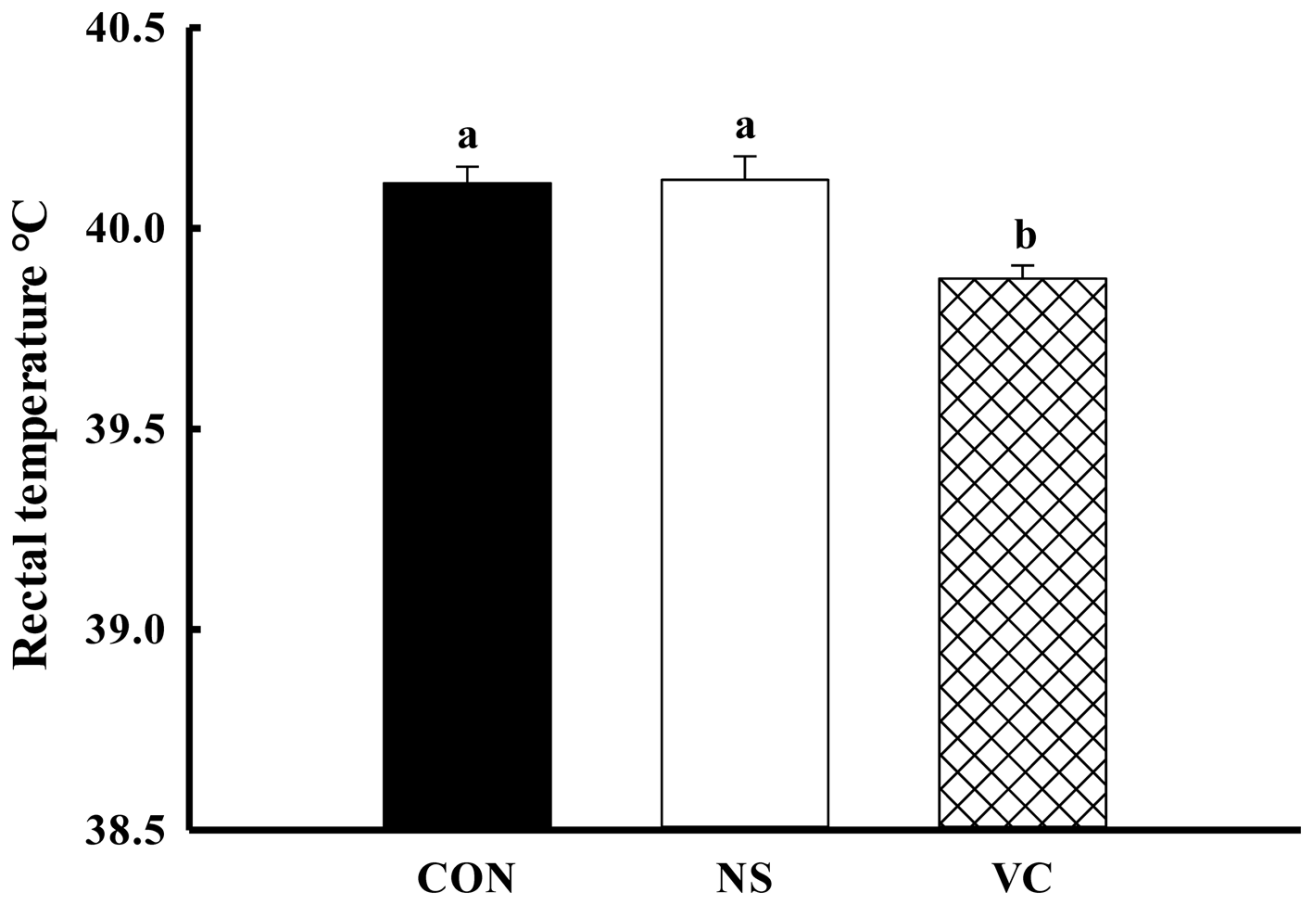
Effects of *in ovo* feeding of vitamin C on rectal temperature in the newly hatched chicks.

As shown in Figure 1B, the percentages of the early hatched chicks among the CON, NS, and VC groups were 29.31, 12.00, and 33.90%, respectively; the proportions of the early and middle hatched chicks among the CON, NS, and VC groups were 51.72, 42.00, and 38.27%, respectively; and the proportions of the late hatched chicks among the CON, NS, and VC groups were 48.28, 58.00, and 23.73%, respectively. As shown in Figure 5, the chicks’ rectal temperature was lower in the VC group than in the CON and NS groups (*p* < 0.05).

### 3.6 Effect of vitamin C on plasma parameters

As shown in Table 3, in the VC group, the biochemical indicators of ALP, TP, ALB, GLB, TBIL, TBA, UA, and HDL-C were lower (*p* < 0.05) and ALT showed an increasing trend (*p* = 0.059), compared to the NS group,. In the VC group, the hormone level of CORT was lower (*p* < 0.05) and T4 showed a decreasing trend (*p* = 0.096), compared to the NS group,

**Table 3:** Effects of *in ovo* feeding of vitamin C at E11 on plasma parameters in the newly hatched broiler chicks.

Items	Treatments		SEM	*P*-value
	NS	VC		
ALT (U/L)	1.11	2.01	0.436	0.059
AST (U/L)	118.93	108.33	15.550	0.510
ALP (U/L)	425.63	270.50	50.310	0.008
TP (g/L)	16.71	11.80	1.086	<0.001
ALB (g/L)	6.29	4.36	0.507	0.002
GLB (g/L)	10.43	7.44	0.621	<0.001
TBIL (μmol/L)	61.01	40.95	5.106	0.002
TBA (μmol/L)	24.09	14.80	3.191	0.011
BUN (mmol/L)	3.37	3.20	0.270	0.549
UA (μmol/L)	218.75	119.25	37.436	0.019
CRE (μmol/L)	9.58	9.96	1.131	0.737
GLU (mmol/L)	11.16	11.10	0.710	0.928
TC (mmol/L)	8.07	7.05	0.603	0.110
TG (mmol/L)	1.94	1.85	0.203	0.677
HDL-C (mmol/L)	3.40	2.15	0.228	<0.001
LDL-C (mmol/L)	2.13	2.29	0.202	0.434
T4 (nmmol/L)	12.05	9.68	1.291	0.096
fT4 (pmmol/L)	8.70	6.49	1.274	0.113
T3 (nmmol/L)	4.00	4.10	0.335	0.760
fT3 (pmmol/L)	21.69	22.72	1.941	0.609
CORT (ng/mL)	29.39	19.35	3.914	0.030

CON, the control group; NS, the normal saline group; VC, the vitamin C group; ALT, alanine aminotransferase; AST, aspartate aminotransferase; ALP, alkaline phosphatase; TP, total protein; ALB, albumin; GLB, globulin; TBIL, total bilirubin; TBA, total bile acid; BUN, blood urea nitrogen; UA, uric acid; CRE, creatinine; GLU, glucose; TC, total cholesterol; TG, total triglyceride; HDL-C, high-density lipoprotein cholesterol; LDL-C, low-density lipoprotein cholesterol; T4, total thyroid hormone; fT4, free thyroid hormone; T3, total triiodothyronine hormone; fT3, free triiodothyronine hormone; and CORT, corticosterone.

## 4 Discussion

During a short period of 21 days, a fertilized egg eventually turns into a viable chick through a series of developmental procedures, including access to oxygen, diverse energy sources, and the accommodation of metabolic patterns (16). The heart, lungs, and chorioallantoic membrane are the basis for ensuring oxygen supply and gas exchange, while the liver is the center of metabolism and the basis for accommodating metabolic patterns. In addition, bile in the gallbladder improves the utilization of lipids, which are the major energy-supplying substances from the yolk. Interestingly, a synchronized progression is observed in the organ indexes of the heart, liver, lungs, and gallbladder, as well as in the relative weight of the embryo (or chick) and residual yolk, which may together reflect embryonic developmental characteristics.

The chorioallantoic membrane forms from E4, gradually develops blood vessels, and attains mature gas exchange capacity by E11-12 (17). Meanwhile, the paired primordia of the heart begin to fuse and develop from E2 (13), and the heart adapts to mature chorioallantoic respiration by E11-12. Nevertheless, the lungs develop capillaries by E18, and pulmonary respiration occurs by E19, gradually replacing chorioallantoic respiration (18). At the same time, the heart is forced to adapt to the transition from chorioallantoic respiration to pulmonary respiration. In summary, E18 is a key time for the transition from chorioallantoic respiration to pulmonary respiration, as well as for cardiac adaptive changes. Therefore, the turning points of the heart and lung indexes were observed at E17 and E15, respectively, indicating that the transition from chorioallantoic respiration to pulmonary respiration forced adaptive changes in the heart.

The liver is structurally completed by E14 and then fully functions as the metabolic center (16), which were synchronized with the increase and stability of the liver index from E11 to E15 and from E15 to E21, respectively, in this study. From E21 to D1, a significant increase in the liver index was attributed to the rapid deposition of lipids in the liver (19). The gallbladder formed at E9 and then grew rapidly, which was consistent with the increase in the gallbladder index from E11 to D1. Bile, secreted by the gallbladder, is beneficial for the liver to utilize lipids from the yolk, which was supported by the relative weight of the residual yolk from E20 to D1. A shift in the growth rate was observed at E13, which may be related to the structural completion of the liver and bile secretion from the gallbladder, both of which together ensure the utilization of lipids as the main energy supply.

Changes in the organ indexes (the heart, liver, lungs, and gallbladder) and the relative weight of the embryo or remaining yolk are synchronized with changes in important physiological functions during embryonic development and can be used as indicators to reflect the hatching characteristics of broiler chickens. Recently, the incubation period accounts for more than 50% of an Arbor Acres broiler’s productive life. Therefore, it is necessary to encourage positive effects on broiler growth performance during the early growth period (20). In this study, IOF of VC had no harmful effects on the relative weight of the embryo (or chick), residual yolk, and organ indexes. In previous studies, it was found that IOF of VC had no significant influence on the weight of D1 chicks, which was the same as our results (7).

At E19, the structural development of the chicken embryo is complete. Before hatching, the embryo must complete hatching behaviors such as absorbing the remaining yolk sac into the abdominal cavity, pecking the shell membrane, and pecking the shell. Early or delayed occurrence of the above behaviors will affect the quality of chicks and the hatching time. Therefore, the hatching time and the score for yolk absorption and the rupture of the shell membrane were selected to comprehensively evaluate the effects of IOF of VC on the hatching process in broilers. In this study, the rupture of the shell membrane and yolk absorption occurred earlier, and the hatching time was correspondingly advanced in the VC group, indicating that VC accelerated the hatching process of preparation for emergence. The hatching muscle, a pair of muscles located at the back of the head, assists in the movement of the embryonic head and mouth (13) and is responsible for puncturing the shell membrane and swallowing amniotic fluid through vigorous contractions (21). The swallowing of amniotic fluid directly facilitates intestinal development (intestinal morphology, digestive enzyme activities, and nutrient transporter expression) and provides the foundation for digestion and absorption of the remaining yolk (22), which is in favor of yolk absorption. We speculated that VC may promote energy reserves in the hatching muscle to accelerate the hatching process, which needs to be further investigated.

Producing chicks with strong anti-stress ability is crucial for the poultry industry. VC has been used to mitigate heat stress by improving production performance through antioxidant modulation in chickens (23). Rectal temperature is a reliable indicator for evaluating anti-stress ability (14, 24), as are stress-related hormones, including CORT (25). CORT is a sensitive indicator in broiler chickens that reflects their physiological condition under stress. To a certain extent, environmental changes before and after hatching are stressful to newly hatched chicks. In this study, IOF of VC reduced the chicks’ rectal temperature and plasma CORT levels, indicating that the chicks in the VC group possessed stronger anti-stress ability. Our previous research showed that IOF of VC could regulate embryonic endogenous protection (heat shock protein) and metabolism, which may reduce the risk associated with overheating in chicken embryos (26). In a previous study, IOF of VC reduced the rectal temperature of newly hatched chicks, but the study lacked a non-injected group (7). This study addressed the shortcomings and further confirmed the results. In addition, some studies have shown that dietary VC could reduce rectal temperature and improve heat tolerance in poultry under heat stress (27).

During the preparation for emergence, dramatic physiological and metabolic changes occur. Any intervention can markedly affect the embryonic hatching process and postnatal performance. In this study, the CON group was set up to confirm the effects of IOF of VC on embryonic development, the hatching process, and the chicks’ rectal temperature, while the NS group served as the strictly biological control group for the VC group. Therefore, the plasma biochemical indicators and hormone levels, sensitively reflecting embryonic physiology and metabolism, were only detected between the NS and VC groups.

It is noteworthy that the liver of newly hatched chicks was rich in lipids (28). Lipids in plasma are mainly derived from egg yolk, which is mainly composed of TG, phospholipids, and cholesterol. IOF of VC decreased the plasma HDL-C levels, implying a reduced transport of cholesterol from the blood to the liver. In addition, IOF of VC decreased the plasma levels of ALP, TBIL, and TBA, indicating that the function of the liver was in better status in the VC group. In summary, IOF of VC may be conducive for the liver to manage the physiological characteristics of rich lipids. The decreased level of plasma UA indicated that VC reduced protein catabolism. Combined with the decreased levels of plasma TP, ALB, and GLB, we speculated that VC promoted protein anabolism. Plasma GLU is mainly regulated by liver glycogen, which is derived from gluconeogenesis from glycogenic amino acids and glycerol during late incubation (29). Based on the reduced protein catabolism, we speculated that VC may promote gluconeogenesis from glycerol in the liver, which may be related to improved liver function.

Thyroid hormones including T3, fT3, T4, and fT4 regulate physiology and metabolism in birds. In addition, thyroid hormones vary dramatically during late incubation and after hatching (30). These hormones play an important role in the thermoregulation of broiler embryos and broiler chickens, involving metabolic heat production (31). In this study, IOF of VC showed a decreasing trend in the plasma T4 level, indicating that the D1 chicks had lower metabolic heat production, which was supported by the lower rectal temperature in the VC group.

## 5 Conclusion

The rupture of the shell membrane and yolk absorption occurred earlier, and correspondingly, the hatching time was advanced in IOF of VC (3 mg/egg), indicating that VC accelerated the hatching process. In addition, IOF of VC improved the anti-stress ability of the D1 chicks, as evidenced by lower rectal temperatures and plasma CORT levels. These effects may be related to the regulation of body energy metabolism.

## Data Availability

The original contributions presented in the study are included in the article/supplementary material, further inquiries can be directed to the corresponding author.
